# Promoting Best Practice in Cancer Care in Sub Saharan Africa

**DOI:** 10.3389/fmed.2022.950309

**Published:** 2022-07-06

**Authors:** Karishma Sharma, Shahin Sayed, Mansoor Saleh

**Affiliations:** ^1^Clinical Research Unit, Aga Khan University Cancer Center, Aga Khan University, Nairobi, Kenya; ^2^Department of Pathology, Aga Khan University Hospital, Nairobi, Kenya; ^3^Department of Hematology and Oncology, Aga Khan University Hospital, Nairobi, Kenya

**Keywords:** low- and lower-middle-income countries, tumor microenvironment (TME), research development-innovation, diversity and inclusion, collaboration pathology and oncology

## Abstract

Promoting best practice in the management of a cancer patient is rooted in the application of new knowledge derived through various sources including population science, laboratory advances, and translational research. Ultimately, the impact of these advances depends on their application at the patient's bedside. A close collaboration between the oncologist and the pathologist is critical in underwriting progress in the management of the cancer patient. Recent advancements have shown that more granular characteristics of the tumor and the microenvironment are defining determinants when it comes to disease course and overall outcome. Whereas, histologic features and basic immunohistochemical characterization were previously adequate to define the tumor and establish treatment recommendation, the growing capability of the pathologist to provide molecular characterization of the tumor and its microenvironment, as well as, the availability of novel therapeutic agents have revolutionized cancer treatment paradigms and improved patient-outcomes and survival. While such capacity and capability appear readily available in most developed high-income countries (HIC), it will take a concerted and collaborative effort of all stakeholders to pave the way in the same stride in the low and middle-income countries (LMIC), which bear a disproportionate burden of human illness and cancers. Patients in the LMIC present with disease at advanced stage and often display characteristics unlike those encountered in the developed world. To keep stride and avoid the disenfranchisement of patients in the LMIC will require greater participation of LMIC patients on the global clinical trial platform, and a more equitable and affordable sharing of diagnostic and therapeutic capabilities between the developed and developing world. Key to the success of this progress and improvement of patient outcomes in the developing world is the close collaboration between the oncologist and the pathologist in this new era of precision and personalized medicine.

## The Critical Interplay Between Pathology and Oncology

There is no better example of the critical interconnectedness between the pathologist and the clinician than in the field of oncology. Decades ago, the primary support function of the pathologist working with the oncologist was in the diagnostic identification and characterization of an identified malignancy. Categories were rather broad, e.g., carcinoma; sarcoma; myeloma; leukemia; and lymphoma (1) with additional features allowing carcinomas to be categorized into adenocarcinoma vs. squamous cell carcinoma (2). The organ of origin was often deduced based on the histologic features of the tumor on H.E. Stain and supporting clinical as well as radiologic features (3). Advances in immunohistochemistry and the identification of key surface markers have enabled a more nuanced characterization of tumors enabling site of origin as well as well as cellular markers of proliferation that add prognostic significance (4). Advances in molecular testing has added a new layer of capability to identify unique molecular characteristics of the tumor that in turn can provide clues into the molecular mechanism of the malignancy as well as point toward potentially effective therapies based on such predictive markers (5).

More recently, with the advent of NextGen Sequencing (NGS) pathologists have the capability of providing the oncologist critical actionable information directly relevant to the treatment and prognosis of the patient with cancer (6–8). Such advances have shored up the critical interconnectedness between the pathologist and the oncologist. This interplay is best observed at the tumor boards where peer discussion and treatment recommendation is heavily dependent on the contribution of the pathologist, wherein therapeutic decision making is based not just on the histologic features and immunohistochemical markers but in addition on predictive molecular characteristics of the tumor (9).

## Importance of the Tumor Microenvironment (TME)

Stromal processes play a critical role in cancer biology and in some cases, may predict the clinical course of a malignancy better than cellular characteristics of the neoplastic cells itself (10, 11). The tumor microenvironment is a dynamic network that includes the tumor cells, stromal tissue within which are the immune cells, fibroblasts, pericytes, cytokines, and tumor vasculature and the extracellular matrix (12). In addition to epithelial and stromal compartments, the tumor microenvironment contains several cell types of the innate and adaptive immune systems including B and T lymphocytes, dendritic cells, and macrophages (13). This tumor microenvironment (TME) provides added dimension that influences both the biology of the tumor as well as its response to therapeutic efforts and the development of resistance (14). It is integrally involved in nearly all processes critical to the malignant process -tumor initiation, growth, migration, metastasis, and therapeutic resistance (15, 16). As an example, Denkert and colleagues (17) were the first to demonstrate that a high Tumor Infiltrating Lymphocyte (TIL) count in breast cancer tissues was associated with a significant pathologic complete remission (pCR) rate following Neoadjuvant therapy compared to TIL low tumors. Higher levels of TILs also predict for increased responsiveness to chemotherapy independent of estrogen receptor (ER), progesterone receptor (PR), and Human Epidermal Growth Factor Receptor (HER2), with each 1% increment associated with a further increase in the rate of pCR (18).

The role of the pathologist has thus evolved beyond the characterization of the tumor itself and moved on to include the non-clonal cellular microenvironment surrounding the tumor.

## Immunotherapy and the Role of the Pathologist

Immunotherapy has become an indispensable arm of cancer management with novel T-cell targeting agents approved by the FDA across various indications both as single agents or in combination with conventional cytotoxic agents (19).

The Nobel Prize in Medicine or Physiology awarded in 2018 acknowledged the important contributions of **James Allison and Tasuku Honjo**, for their pioneering work on cancer immunotherapy (20). Hailed as a revolution in the treatment of cancer, immunotherapy works by boosting the body's natural defenses against cancer. The Nobel committee heralded the efforts of the two scientists as establishing an entirely new principle for cancer therapy by stimulating the inherent ability of our immune system to attack tumor cells (21, 22).

These advances could not have been possible without the investigative role of the pathologist giving importance to not just the tumor cells but to the surrounding TME and tumor infiltrating T-cells (23). The role of the pathologist in investigating the TME in an objective reproducible way is best exemplified by the Programmed Cell Death Ligand combined positivity score (PDL1-CPS)which has become critical as a companion diagnostic tool for the use of a number of novel immunotherapy agents approved by the FDA (24). In the years to come the need for tumor NGS and TME in forging new therapeutic advances will become increasingly evident and critical (25).

## Closing the Gap- Access to Molecular Diagnostics

The recent advances in molecular diagnostics have contributed to an increasing disparity between patients in the West and those in the developing world where access to such technology remains wanting, both because of lack of trained pathologists with molecular sub-specialization but also as a result of lack of testing facilities within sub Saharan Africa (26, 27). One solution toward bridging this gap is the establishment of collaboration between pathology departments in the LMIC with molecular diagnostic laboratories in the West and the creation of virtual molecular tumor boards both as a platform for peer discussion and capacity building (28). Part of the impetus for such collaboration may well be the heightened interest in the molecular characterization of cancers diagnosed in the African continent (29). As part of the research collaboration, institutions in SSA could receive access to subsidized Next Generation Sequencing (NGS) of their tumor samples, participation on virtual molecular tumor boards where such cases can be discussed, as well as, opportunity for the patients to participate on clinical trials (30). An important component that would effectively bridge this divide and result in actionable benefit to the African patient would be the availability of novel therapeutic agents for the treatment of patients identified to harbor unique actionable molecular features. Therein lies the opportunity to enroll such patients onto clinical trials that offer access to novel treatments that would otherwise not be available to such patients.

## Multidisciplinary Tumor Boards and Specialty Clinics

Tumor boards represent an ideal platform for data driven peer discussion, both as a means to establish best practice for common cancers, but also as a venue for the multi-disciplinary discussion of complex cases in the context of existing resource limitations (9). The incorporation of peer partners in virtual MDTs from Western academic centers provides an important avenue for sharing new knowledge as well as identifying areas of need for diagnostic investment in the LMIC (31, 32). In addition, such platform provide the opportunity for peer discussion and knowledge transfer that ultimately making the optimal therapeutic choice for the patient.

Beyond the multidisciplinary tumor boards, since January 2021, we have moved to establish specialty clinics dedicated for the multi-disciplinary in-person consultation of patients with specific cancers at the Aga Khan University Hospital Nairobi (AKUHN). We piloted our project with patients presenting with newly diagnosed breast cancer (33). The success of this program resulted in the establishment of a similar platform for patients with newly diagnosed prostate cancer.

The multi-disciplinary in-person clinics go one step beyond the tumor boards and allow the patient to be seen by a team of specialists, including their own primary oncologist. In the case of the multi-disciplinary breast cancer clinic (MBC) and multi-disciplinary prostate cancer clinic (MPC), the consultant team includes a medical oncologist, breast surgeon or urologist, radiation oncologist, and a team of nurses and coordinators. The patient gets to be interviewed and examined by an independent physician member of the team, who then presents the case at the “huddle” where the tumor board discussion and recommendation is revisited in the context of the patient's own input. The management decision from the “huddle” is then conveyed to the patient and family who then have the opportunity to ask questions relating to the entire management plan.

**Figure 1:**
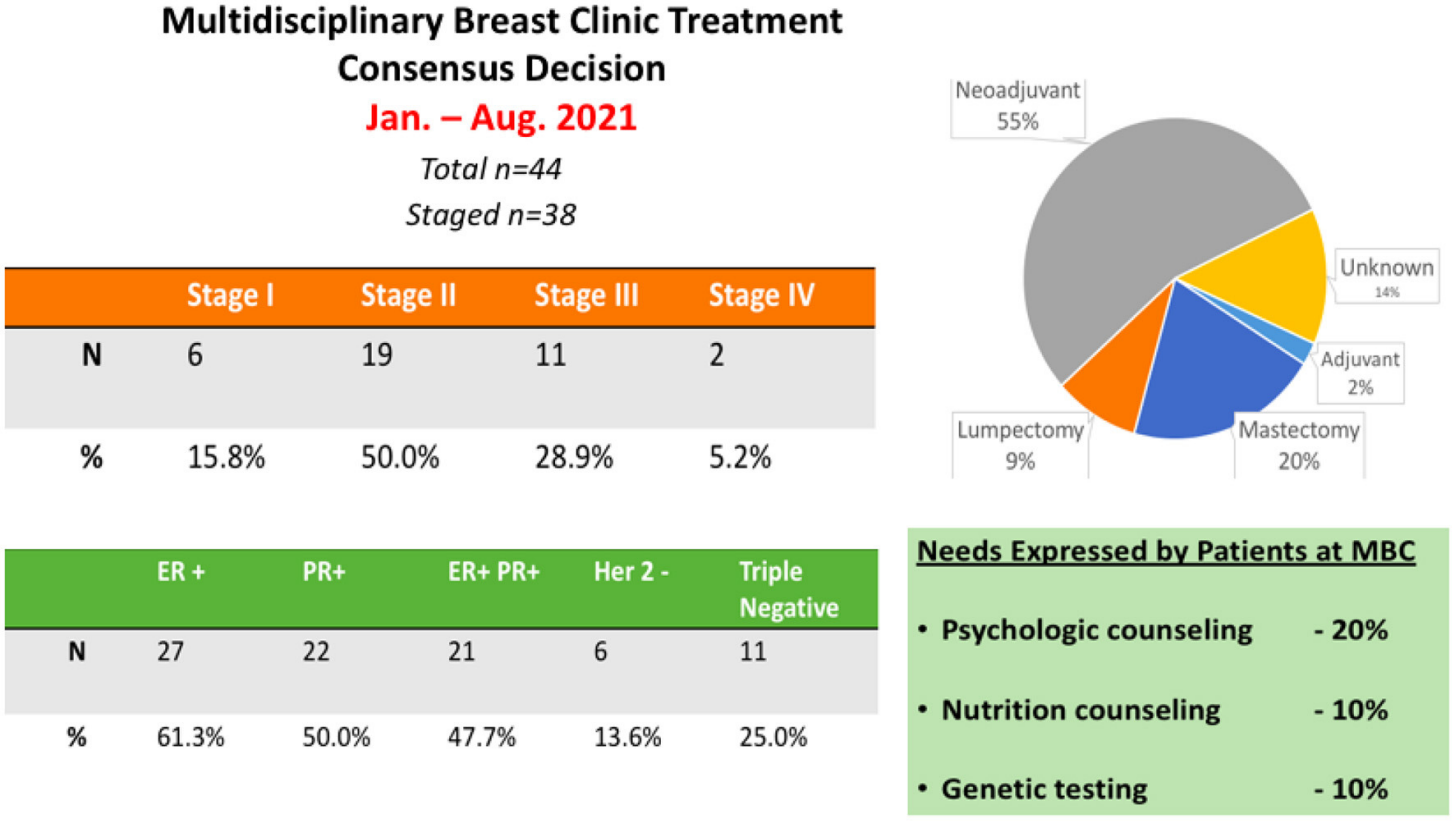
Summary of the data collected from the MBC clinics at AKUHN over a period of 6 months.

Our experience at the Aga Khan University Hospital Nairobi has demonstrated an enthusiastic embrace of the MBC and MPC model. Interestingly, the multidisciplinary engagement with the patient provides a novel platform to discuss all aspects of the patient management journey in one setting. Our experience at AKUHN has demonstrated that >90% of patients and family members wish to participate in a multi-disciplinary consultation that is provided at no extra charge. Patients get to voice their input and buy-in for the proposed treatment plan. Interestingly, in 20% of the cases, patient preferences result in modification of the treatment plan both in terms of surgical management or selection of neoadjuvant vs. adjuvant therapy. In 10% of cases, patients have desired additional psycho-social counseling and in 10% of cases patients requested nutritional and dietary counseling. A summary of the data collected from the MBC clinics at AKU is provided (Figure 1). This platform also serves as an ideal setting to inform patients about clinical trials and to initiate the informed consent process for ongoing research studies including clinical trials.

## Promoting Cancer Clinical Trials in SSA

Africa makes up 1/6 of the world's population (34). Of the 2.7 million clinical trials conducted internationally, less than a fraction of 1% are conducted in the African continent with participation of African subjects (35). This disparity was further unmasked during the Covid-19 pandemic when <2% of the clinical trials linked to the pandemic were conducted in the African continent (36) and African patients had limited access to novel treatment approaches such as Tocilizumab, Remdesivir or participation in convalescent plasma studies. Within Africa itself, nearly 2/3 of the clinical trials are conducted either in Egypt or South Africa with marginal presence in the remaining 44 countries within the continent (35). In the year 2019, of a total of 109 oncology clinical trials initiated in Africa, most of these were conducted in North or South Africa and out of which there were only 6 conducted in Sub Saharan Africa sites (37).

The African continent has long been the platform for studies investigating the prevention and treatment of communicable diseases such as malaria, tuberculosis, HIV etc. However, with the rise in the life expectancy across the continent and lifestyle changes, we have witnessed a rise in the incidence and mortality associated with non-communicable diseases, specifically cancer (38). Establishing clinical trial centers in SSA with well-trained and experienced staff can provide an ideal platform to conduct cutting edge clinical trials in the African continent (39, 40). Conducting therapeutic oncology trial not only levels the playing field for patients in the African continent, provides patients in SSA access to novel therapeutics and allows staff to be trained and experience the unique toxicity profile of such molecules. For the pharmaceutical industry this provides the opportunity to study a patient population with a vastly different ethnic and genetic background, which may well-influence both the toxicity as well as the outcome of such therapeutic agents. Genetic diversity and its impact on drug metabolism has long been demonstrated and may well-impact how African patients tolerate and respond to such novel anti-cancer therapies (41).

“There is increasing recognition of the need for pathologists to be involved early in trial planning and design to ensure methodological rigor in trials requiring sample collection, procurement, storage, transportation and analysis” (42). The recommendations for Interventional Trials 2013 Statement has recently been expanded to include Pathology (SPIRIT-Path) (43). “The guidelines allow investigators to comprehensively address the cellular and molecular pathology aspects of trial protocols, ensuring adequate skills and resources are available at trial commencement and fully leverage the value of biospecimens for translational research” (43).

## Understanding the Role and Contribution of Genetic Diversity

Genetic variations may also be critical in determining effective strategies for the diagnosis and treatment of cancers in patients of African origin given increasing evidence of the molecular diversity of tumors found in African patients as well as a different risk profile as compared to their N. American counterparts (29, 44). As an example, a study of triple negative breast cancer comparing mRNA signatures of women diagnosed with triple negative breast cancer in African women from Kenya revealed a differential expression of both up-regulated as well as down-regulated genes as compared to African American (AA) and Caucasians women from Alabama (45). Similarly, another study comparing AA and European Americans (EA) revealed clear differences in tumor biology between the two groups (46). A recent study looking at AA and EA found significant genetic difference in patients with prostate cancer which may potentially contribute to the incidence and outcome of prostate cancer in these populations (47). It is thus critical that the development of novel therapeutics especially in oncology include the participation of African patients in drug development trials. A current low enrollment of 4% of minority patients in clinical trials conducted in N. America could well be supported and augmented if clinical trial sites in Africa were included (48).

A number of actions would have to be taken if Africa is to become a major partner in drug development. Recent data from the World Bank suggests that African countries invest <1% of their GDP in research and development as compared to USA that spends close to 3% (49). The absence of universal health coverage, the lack of awareness and near absence of cancer screening initiatives and inability to afford standard diagnostic procedures contributes significantly to late presentation and early death among African patients with cancer (38).

## Bridging the Gap

Of the 54 countries in Africa only 25 countries have functional population based cancer registries through the AFCRN (African Cancer Registry Network) program that was set up in the year 2012 (50). For decades, the burden, pattern and outcome of cancer in Africa has been understudied. Even functional registries face countless challenges that contribute to the data being inaccurate and unrepresentative. These include generally poor health care infrastructure, lack of a regular and accurate census program' absence of vital statistics, lack of adequately trained personnel and lack of cooperation/contribution from other data sources (51). Computer-based medical information systems also remain underdeveloped (51). Researchers with experience conducting studies in Africa have opined on the following barriers: lack of infrastructure, financial and human capacity, delays in regulatory and ethical reviews, complex logistical and financial systems (52).

Another major hurdle facing SSA is the lack of training of health care workers in the field of research. Out of 909 training institutions in Africa, only 20% offer training in clinical investigation and research. All of these trainings are at post graduate level and research is not part of the curriculum at the undergraduate level. The vast majority (over 50%) of the training institutions are in Kenya, Ethiopia, SA and Ghana (53).

Despite the large human population and disproportionate burden of disease, Africa lacks the human resources necessary to implement an effective cancer control program. Only 7 African countries have a pathologist to population ratio of more than 1/1,000,000. This is in contrast to 1 pathologist per 15–20,000 population in Europe and N. America (54). A shortage of oncologists also exists in over 25 countries in Africa with clinical oncologist to new patient ratios exceeding >1,000 compared to <150 in the west. It is also astounding to note that ~8 countries in Africa including Sierra Leone, Burundi and Togo have no clinical oncologists (55). At this stage, unless the paradigm changes, it would take Africa over 400 years to catch up (54). In addition, this disparity is further compounded by the lack of sophisticated molecular diagnostic and treatment capabilities even in those countries that have well-trained pathologist/Oncologists.

In the last decade there have been a number of initiatives by pharmaceutical companies and western nations to establish projects to improve the research capacity, infrastructure and collaboration within SSA (56). Establishment of clinical trials cooperative groups such as those in N. America and Europe will be key to allowing Africa to leap frog and catch up with Western partners. It remains crucial for governments, NGO and clinicians themselves to invest efforts and funds toward the establishment of a more robust cancer prevention and control program in SSA.

As oncology moves from a one size fits all to a more nuanced and personalized approach employing precision medicine technology, the role of the pathologist and interconnectedness with the oncologist, and the close interplay between the pathology bench and the clinical bedside will become increasing important. The pathologist is no longer just a distant consultant involved with the tissue obtained at surgery. Nor is the modern oncologist one who limits him or herself to interpreting the pathology report and prescribing a treatment regime. There is a growing need for the pathologist to become involved at the bedside and understand the nature of the malignant illness, and for the oncologist to better understand and interpret clinical relevance of the technologies applied at the pathology bench. The treatment of the cancer patient of the future will depend on the clinical findings at bedside, interpretation of sophisticated diagnostic studies, and the translation of modern molecular diagnostics results. The successful treatment of the cancer patient will depend heavily on the interconnectedness between the pathologist and oncologist and the availability of novel therapeutic, aided by the availability of clinical trials.

## Data Availability Statement

The original contributions presented in the study are included in the article/supplementary material, further inquiries can be directed to the corresponding author.

## Author Contributions

MS was involved with the conceptualization. KS and MS was responsible for writing and editing the original draft. SS was involved with reviewing and editing the final draft. All authors contributed to the article and approved the submitted version.

## Conflict of Interest

The authors declare that the research was conducted in the absence of any commercial or financial relationships that could be construed as a potential conflict of interest.

## Publisher's Note

All claims expressed in this article are solely those of the authors and do not necessarily represent those of their affiliated organizations, or those of the publisher, the editors and the reviewers. Any product that may be evaluated in this article, or claim that may be made by its manufacturer, is not guaranteed or endorsed by the publisher.
